# Conductance modulation of charged lipid bilayer using electrolyte-gated graphene-field effect transistor

**DOI:** 10.1186/1556-276X-9-371

**Published:** 2014-07-30

**Authors:** Mohammad Javad Kiani, Fauzan Khairi Che Harun, Mohammad Taghi Ahmadi, Meisam Rahmani, Mahdi Saeidmanesh, Moslem Zare

**Affiliations:** 1 Faculty of Electrical Engineering, Universiti Teknologi Malaysia, Skudai, Johor 81310, Malaysia; 2 Department of Electrical Engineering, Islamic Azad University, Yasooj branch, Yasooj 75916, Iran; 3 Department Of Electrical Engineering, Urmia University, Urmia 57147, Iran; 4 Department of Physics, Yasouj University, Yasouj 75914-353, Iran; 5 School of Physics, Institute for Research in Fundamental Sciences (IPM), Tehran 19395-5531, Iran

**Keywords:** Monolayer graphene, Conductance modulation, Lipid bilayer, Electric charge

## Abstract

Graphene is an attention-grabbing material in electronics, physics, chemistry, and even biology because of its unique properties such as high surface-area-to-volume ratio. Also, the ability of graphene-based materials to continuously tune charge carriers from holes to electrons makes them promising for biological applications, especially in lipid bilayer-based sensors. Furthermore, changes in charged lipid membrane properties can be electrically detected by a graphene-based electrolyte-gated graphene field effect transistor (GFET). In this paper, a monolayer graphene-based GFET with a focus on the conductance variation caused by membrane electric charges and thickness is studied. Monolayer graphene conductance as an electrical detection platform is suggested for neutral, negative, and positive electric-charged membrane. The electric charge and thickness of the lipid bilayer (*Q*_LP_ and *L*_LP_) as a function of carrier density are proposed, and the control parameters are defined. Finally, the proposed analytical model is compared with experimental data which indicates good overall agreement.

## Background

**Figure 1:**
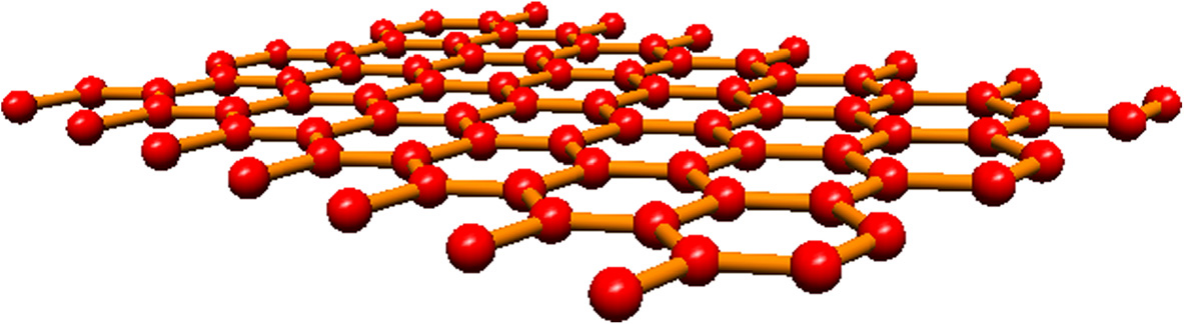
Monolayer graphene structure with one-atom thickness.

Graphene is a monolayer of sp^2^-bonded carbon atoms, and this sp^2^ bond makes the graphene structure look like honeycomb crystal, as shown in Figure 1[1]. Graphene is called the mother of graphite (many layers of graphene) because it can act as the basic building block of these allotropes [2,3]. Graphene was theoretically discovered back in the 1940s, but at that time, graphene (a 2D layer crystal) was believed to be too thermodynamically unstable to be produced in the real world [4].

After Andre Geim and Konstanstin, Novoselov successfully produced graphene from Scotch tape in 2004, research attention has moved forward rapidly on graphene.

**Figure 2:**
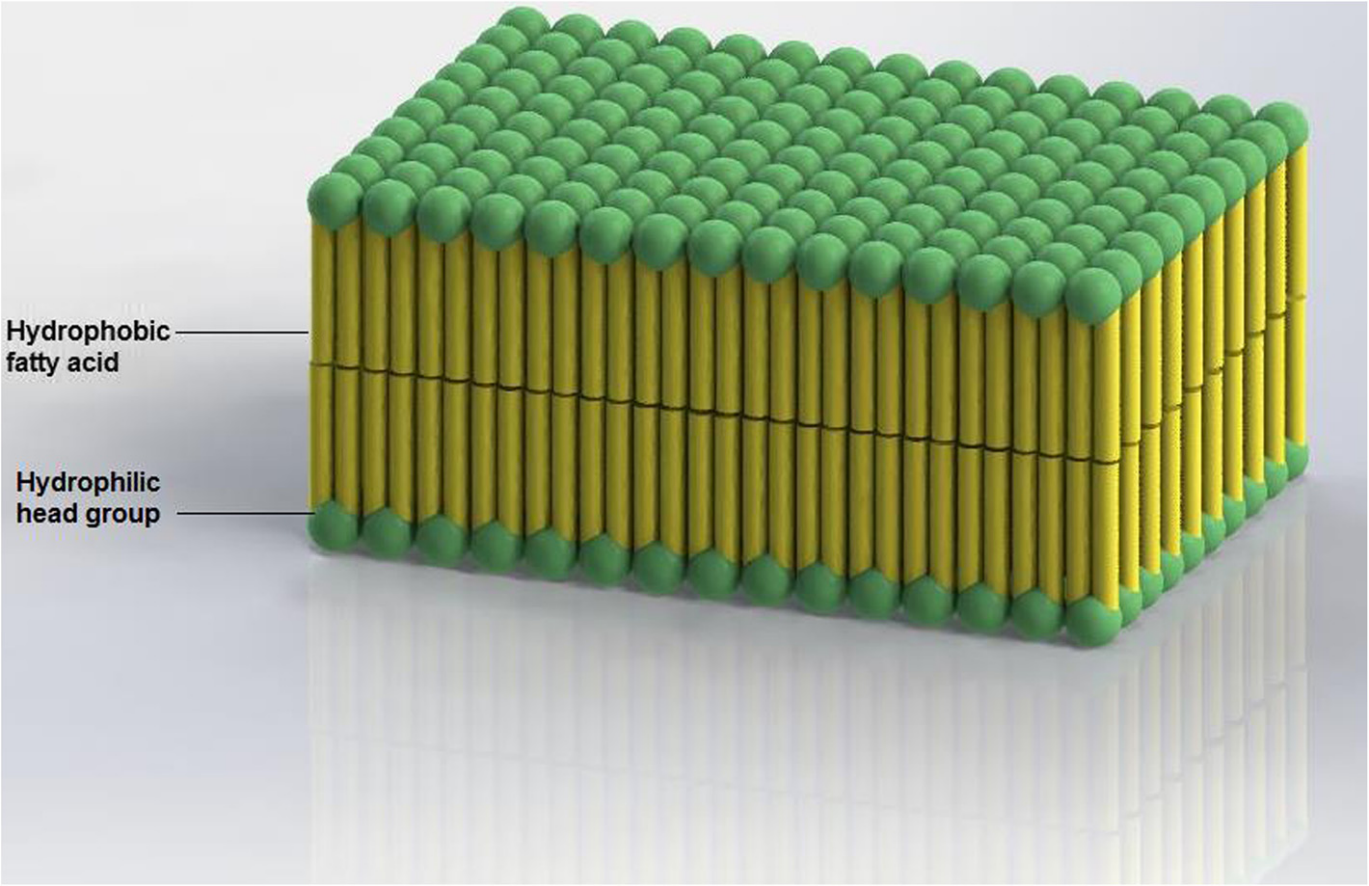
Structure of phospholipid bilayer.

Graphene has attractive electrical properties such as the ability to continuously tune the charge carriers from holes to electrons, high mobility, and high-carrier velocity [5-8]. The charge carrier (electron) in graphene can be explained by electron propagation through the honeycomb lattice of graphene that develops after the electrons lose their effective mass, which yields quasi-particles called ‘Dirac fermions’ [9]. These Dirac fermion particles are hard to imagine because they have no known analogies [9]. They can be illustrated by a combination of both Dirac and Schrödinger equations. In addition, graphene requires current to be effective, precise, and faster than any other metal on biosensors, in the same way as a biomimetic membrane-coated graphene biosensor [10]. Several types of animal and plant cells are surrounded with a two-layer covering, which is called the phospholipid bilayer [11]. As shown in Figure 2, the molecules that make up the phospholipid bilayer, called phospholipids, organize themselves into two corresponding layers, shaping a covering that can only be infiltrated by certain kinds of substances [11]. This gives the cell an apparent barrier and keeps useless materials out [12].

Although the phospholipid bilayer frequently works well, it can be damaged, and some superfluous materials can penetrate it. Phospholipids have two ends; the first is hydrophilic and attracts water; and the second is hydrophobic and resists water [12]. As the inside of the cells is typically water and the region outside the cells is generally water, these molecules organize themselves into two sheets, with the hydrophilic ends of each layer pointing outwards and the hydrophobic parts pointing inwards [1]. While they are fats or lipids, they are not crushed by the water and are firm enough to prevent large molecules passing through without the assistance of some other material [1].

Some smaller molecules, such as carbon dioxide and oxygen, can pass through without difficulty on their own, but larger molecules such as water, sodium, or magnesium cannot pass easily [13]. The interior of the membrane is also liquid, and this lets proteins, cholesterol, sphingolipids, or sterols converge in it. The role of sphingolipids is to protect the outside of the cell, and the role of the sterols and cholesterols is to stabilize the phospholipid bilayer in plant and animal cells, respectively [13]. Although this is critical for cells to have enough constancy, a large amount of cholesterol can make them inflexible, which is hazardous especially if they are part of a vein that must be flexible to allow blood flow [10]. The proteins are used to transfer materials in or out of the cell throughout the bilayer and to provide places for certain materials to attach to the exterior of the cell [10]. Providing the structure to a cell is one of the major roles of the phospholipid bilayer, which it performs because of the natural arrangement of the hydrophilic and hydrophobic ends of the phospholipids and, once stable, the sterols and cholesterol [10]. Its other role is to control the kinds of materials that can go into the cell or attach to it, which it does in a number of ways using proteins [4]. The kinds of protein that expand from the top of the membrane can be used to recognize the cell or to make a place for specific materials to attach to it [1]. Also, some types of proteins can shape tunnels or channels to allow certain substances to go through. Some channels are always open for certain types of molecules, while others need energy to open and close like gates [14]. This kind of transportation is active transport and can work in both ways, to bring substances in and out of the cell. It is generally used with materials like calcium, potassium, and sodium [15]. A charged lipid bilayer adsorbing on the surface can adopt the electronic properties of graphene. An electrolyte-gated biomimetic membrane-graphene transistor can be used to monitor electrically the bio-recognition events that lead to changes in the membrane's uprightness. Graphene can sense electrically the bactericidal motion of antimicrobial peptides based on a multipart interaction of an ionic screening effect and biomolecular doping [15]. The graphene-based FET structure can be used in the sensing of biological events when there is variation of electrical parameters. The observed transfers of the Dirac point, along with the indication of lipid charges, is an indicator of the charge-impurity potential made by the lipid membranes and shows clearly that the exciting lipid membranes adapt the electronic properties of graphene considerably. Assuming an equivalent division of exciting lipids in the two leaflets, since graphene is an electrically neutral substrate, the concentration of charged pollutants in the lipid membranes can be approximated from the surface area connected to a lipid head group. Also, an analytical modeling for electrolyte-gated biomimetic membrane-graphene biosensor is essential to improve and more recognize the impact of both thickness and electrical charge on the biomimetice membrane. By means of the charged lipid bilayer's adsorption on the membrane surface, the conductance of graphene can be adapted and replicated. Biorecognition actions which cause modifications to the membrane integrity can be considered electrically using an electrolyte-gated biomimetic membrane-graphene biosensor (GFET). In the current paper, a monolayer graphene-based GFET with a focus on the conductance variation caused by membrane electric charges and thickness is studied. Monolayer graphene conductance as an electrical detection platform is suggested for neutral, negative, and positive electric membrane. In addition, the effect of charged lipid membranes on the conductance of graphene-based GFET is estimated regarding the significant shift in the Dirac point in the *G*-*V*_g_ characteristic of the graphene-based biosensor. A monolayer graphene-based GFET with a focus on the conductance variation caused by membrane electric charges and thickness is studied. Monolayer graphene conductance as an electrical detection platform is suggested for neutral, negative, and positive electric membrane. The electric charge and thickness of the lipid bilayer (*Q*_LP_ and *L*_LP_) as a function of carrier density are proposed and the control parameters are defined.

### Proposed model

**Figure 3:**
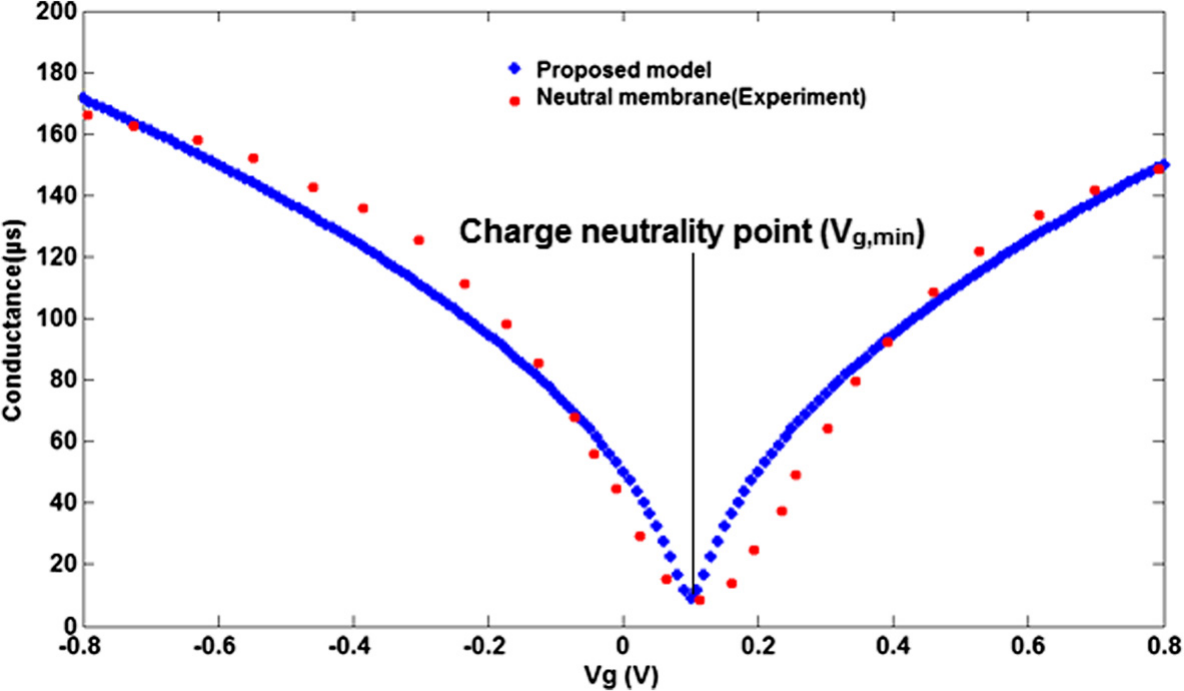
Comparison between bipolar transfer curve of conductance model (blue line) and experimental extracted data (red line) for neutral membrane.

The monolayer graphene in an electrolyte-gated biomimetic membrane graphene transistor with a ballistic channel is assumed to monitor the changes in membrane integrity. High-carrier mobility is reported in experiments on the graphene, which is thought to be due to the totally ballistic carrier transportation in the graphene, which leads to a higher transmission probability. By applying the Taylor expansion on graphene band energy near the Fermi point, the *E* (*k*) relation of the GNR is obtained as [17].

(1) $$E\left(\vec{k}\right)=\pm \frac{3\mathrm{ta}}{2}\sqrt{k_{x}^{2}+\beta^{2}}$$

where *k*_*x*_ is the wave vector along the length of the nanoribbon and *β* is quantized wave vector given by [18]. Based on this wave vector, number of actual modes *M*(*E*) at a given energy which is dependent on the sub bands location can be calculated. By taking the derivatives of wave vector *k* over the energy *E* (dk/dE), the number of the mode *M*(*E*) is written as

(2) $$M\left(E\right)=\frac{\mathrm{\Delta E}}{\mathrm{\Delta k}.L}=\frac{3\mathrm{at}}{2L}{\left(\frac{4E}{3\mathrm{at}}-2\beta^{2}\right)}^{\frac{1}{2}}$$

where *L* is the length of the nanoribbon. A higher transmission probability causes a higher carrier conductance from source to drain, as provided by the Boltzmann transport equation [2,3]:

(3) $$G=\frac{2q^{2}}{h}\int_{-\infty }^{+\infty }\mathrm{dEM}\left(E\right)T\left(E\right)\left(-\frac{\mathrm{df}}{\mathrm{dE}}\right)$$

where *q* is the electron charge, Planck's constant is shown by *h*, *E* is the energy band structure, *M*(*E*) is the number of modes, *f* is the Fermi-Dirac distribution function and *T*(*E*) is the transmission probability. On the other hand, because of the ballistic transport *T*, the possibility of one inserted electron at one end that can be conveyed to other end is considered equivalent to one (*T*(*E*) = 1). The number of modes in accordance with the Landauer formula with respect to the conductance of monolayer graphene can be written as

(4) $$M\left(E\right)=\frac{\propto -3\beta k^{2}}{l}$$

where the length of the graphene channel is shown with parameter *l*, *k* is the wave vector, and $\propto =\frac{\Delta v_{F}}{\surd 2t},$$\beta =\frac{v_{F}^{3}}{\Delta \surd 2t}$. It can be affirmed that the length of the channel has a strong influence on the conductivity function. Taking into consideration the effect of temperature on graphene conductance, the boundary of the integral is changed. This equation can be numerically solved by employing the partial integration method:

(5) $$\begin{array}{l} G=\frac{3q^{2}{\left(3\pi a^{3}t^{3}k_{B}T\right)}^{\frac{1}{2}}}{\mathrm{hl}}\\ \;\times \left[\int_{0}^{+\infty }\frac{X^{-1/2}}{\left(1/\left(1+e^{X-\eta }\right)\right)}\mathrm{dX}+\int_{0}^{+\infty }\frac{X^{-1/2}}{\left(1/\left(1+e^{X+\eta }\right)\right)}\mathrm{dX}\right] \end{array}$$

where *x* = (*E* - *E*_g_)/*k*_B_*T* and the normalized Fermi energy is *η* = (*E*_F_ - *E*_g_)/*k*_B_*T*. Thus, the general conductance model of single-layer graphene obtained is similar to that of silicon reported by Gunlycke [16]. According to the conductance-gate voltage characteristic of graphene-based electrolyte-gated graphene field effect transistor (GFET) devices, the performance of biomimetic membrane-coated graphene biosensors can be estimated through this equation. By assuming that the source and substrate terminals are detained in ground potential, the channel region has the characteristics of the resistor in the small voltage between the source and drain (*V*_DS_). As shown in Figure 3, the performance of a lipid bilayer-based sensor based on graphene nanostructure is assessed by the conductance characteristic. Before the electrolyte solution has been added, pure water as a water-gated ambipolar GFET was added into the membrane to measure the transfer curve. There is substantial agreement between the proposed model of the lipid bilayer-based biosensor and the experimental result which is extracted from the reference [10].

**Figure 4:**
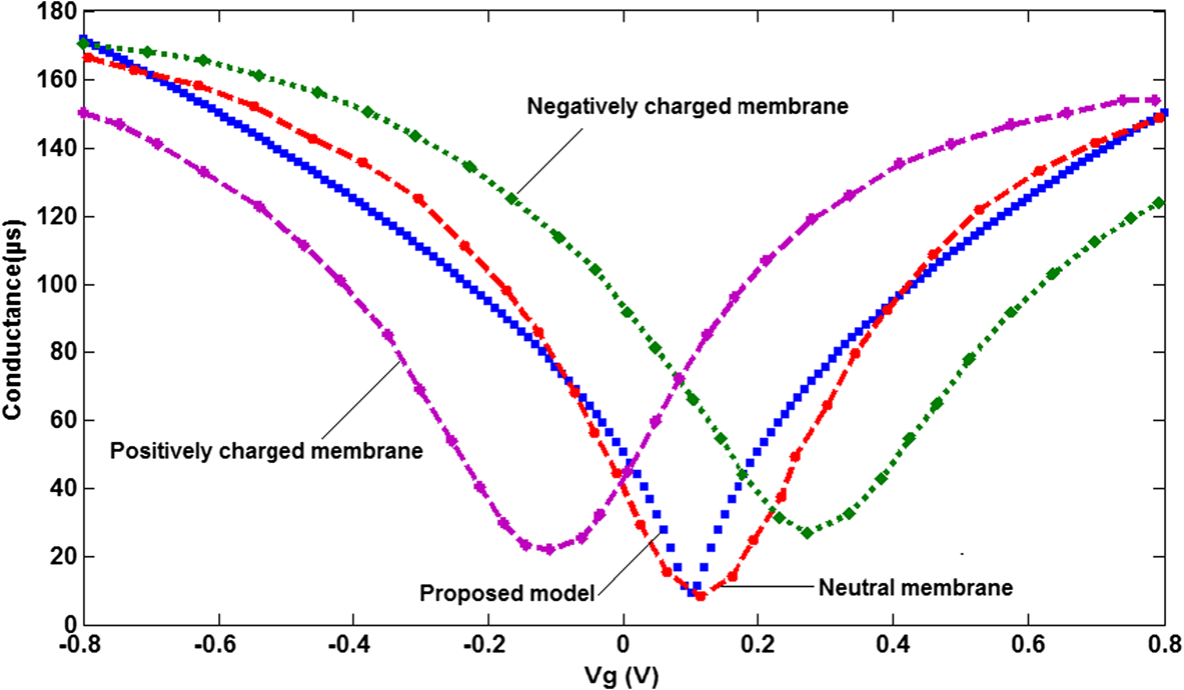
**Comparison between GFET-conductance model and extracted experimental data**[10]**.** For graphene coated with negatively charged, positively charged and neutral POPC membranes.

**Figure 5:**
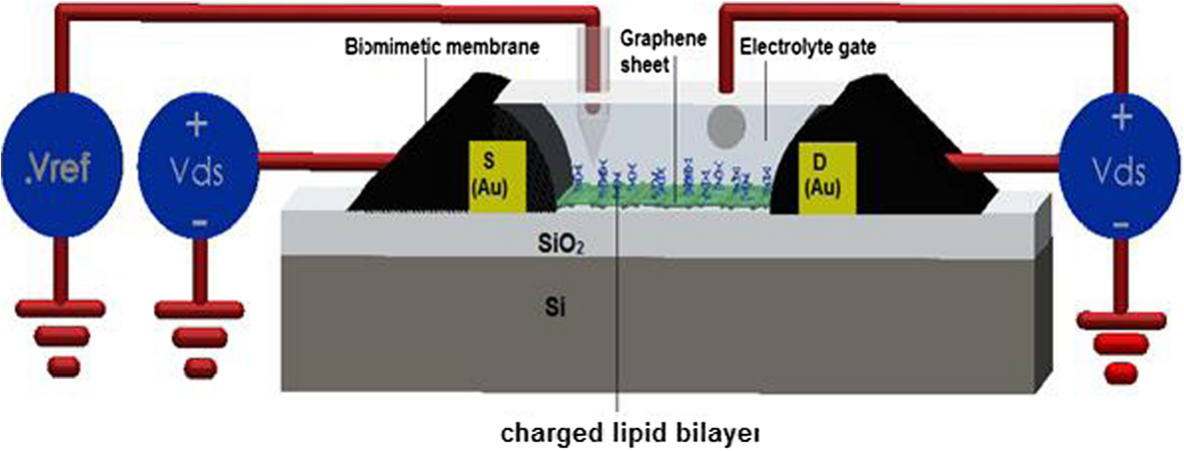
**Schematics of the structure and the electrical circuit of the electrolyte-gated graphene-FET for charged lipid bilayer detection**[10]**.**

**Figure 6:**
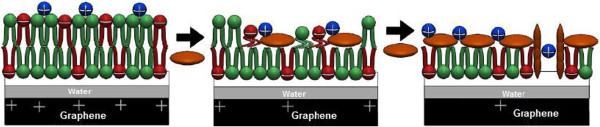
Schematic of lipid bilayer-adsorption processes by surface area of single-layer graphene.

As depicted in Figure 4, by applying the gate voltage to the biomimetic membrane, it is clearly seen that the conductance of GFET-based graphene shows ambipolar behavior. The doping states of graphene are monitored by the *V*_g,min_ to measure the smallest conductance of the graphene layer, which is identified from the transfer characteristic curve. In total, the *V*_g,min_ shift (at the Dirac point) can be considered as a good indicator for lipid bilayer modulation and measurement. Nevertheless, the magnitude of the voltage shift from both positive and negative lipids is comparable when this shift is measured from the position of the minimum conductivity of bare graphene. As shown in Figure 4, the changes in the membrane's electric charge can be detected electrically. The conductivity graph is changed when the electric charges are changing for biomimetic membrane-coated graphene biosensor. So, more electrically charged molecules will be adsorbed and the sensor will be capable of attracting more molecules, which leads to a change in the *V*_g,min_ on the device, and the hole density value can be estimated as decreasing. A negatively exciting membrane demonstrates a very small enhancement in conductivity and a positive change in the Dirac point compared with that of exposed graphene.This is because of an enhancement in the remaining pollution charges caused by the negatively charged membrane. A detection-charged lipid bilayer can be obtained based on a detectable Dirac point shift. In light of this fact, the main objective of the current paper is to present a new model for biomimetic membrane-coated graphene biosensors. In this model, the thickness and the type of coated charge as a function of gate voltage is simulated and control parameters are suggested. Subsequently, to obtain a greater insight into the role of both the thickness and the type of lipid bilayer, GFET modeling is employed to identify the relationship between the conductance and the voltage of the liquid gate, where two electrodes of the sensor, as shown in Figure 5, are considered as the source and drain contacts.The conductance of the GFET channel is dependent not only on the operating voltage on the source-drain channel and graphene organization, but also on the biomimetic membranes of diverse surface charges that accumulate on the graphene and charged lipid bilayer, absorbed by the graphene surface. The conductivity of the graphene-based GFET device is influenced by the charge carrier density changing in the channel. As shown in Figure 6, because of the membrane thinning effect, the conductance of the channel is altered.

Different ions can be adsorbed by changes in the membrane's electric charge and thickness, and subsequently, the sensor will be capable of attracting the ions in the solution which have caused a transformation in the conductance of the graphene-based biosensor. Dependent upon the channel conductance in the biomimetic membrane-coated graphene biosensor, it is concluded that GLP is a function of electric charge and thickness, where GLP is the channel conductance after adding the lipid bilayer. The focus of the present paper is to demonstrate a new model for GFET to measure changes in the membrane's electric charge and thickness. In other words, the conductance of the GFET device as a function of different electric charges and thicknesses is simulated and an electric charge factor (*α*) and thickness factor (*β*) are suggested. Subsequently, for better understanding of the role of the lipid bilayer, FET modeling is employed to obtain an equation describing the conductance, electric charge, and thickness, where the suggested structure of the GFET is shown in Figure 5. This means that *G*_LP_ is considered to be a function of electric charge (*Q*_LP_) as follows. G_*LP*_ = *G*_*Neutral*_ + *αQ*_*LP*_ where electric charge factor (*α*) is assumed, *G*_LP_ is the channel conductance of graphene with biomimetic membranes of different surface charges, and *Q*_LP_ is the electrical charge of the membrane. Consequently, the supposed conductance model of the graphene-based GFET channel can be written as.

(6) $$\begin{array}{l} G_{\mathrm{LP}}=\left(\frac{3q^{2}{\left(3\pi a^{3}t^{3}k_{\mathrm{BT}}\right)}^{\frac{1}{2}}}{\mathrm{hl}}\left[{ℑ}_{\frac{-1}{2}}\left(\eta \right)+{ℑ}_{\frac{-1}{2}}\left(-\eta \right)\right]\right)\\ \;+\left(\alpha Q_{\mathrm{LP}}\right) \end{array}$$

**Figure 7:**
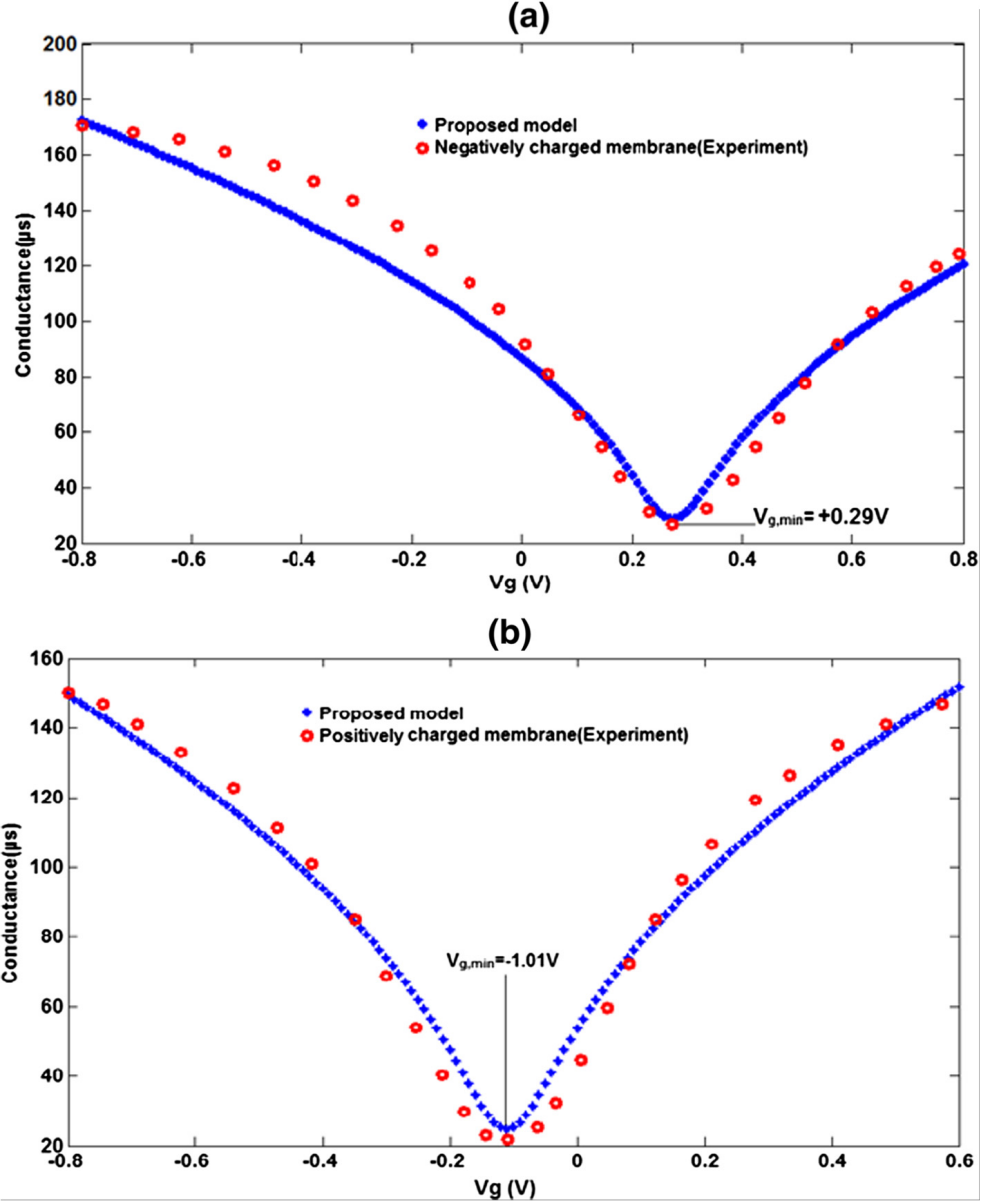
**Comparison between graphene conductance model and extracted experimental data**[10]. **(a)** For negatively electric charges. **(b)** For positively electric charges.

In Figure 7a,b, each diagram clearly depicts the specific electric charge. For example, when graphene is coated with a negative charge, it is noteworthy that the model is closer to the experimental data; in the same manner, we can compare graphene coated with the positive charge as well. It is clearly shown that, by varying the electric charge through the electric charge factor, the *G*-*V*_g_ characteristic curve can be controlled.

Furthermore, the proposed model is strongly supported by the experimental data. According to Figure 7, the amount of *V*_g,min_ shift is changed by changing the electric charge from a negatively charged membrane to a positively charged membrane, probably due to the fact that the changing electric charge has an effect on the Dirac point. It is evident that the graphene channel will be doped to an n-type region with a negatively charged membrane, whereas it changes to hole doping under a positively charged membrane. By increasing the membrane thickness on the graphene surface, the *V*_g,min_ is dramatically left-shifted. It can therefore be concluded that *V*_g,min_ is very sensitive to the electric charge and the thickness of the membrane. To support this, the gate voltage shifted leftwards owing to the fact that the graphene will be n-doped by the high membrane thickness. On the other hand, the conductivity of the graphene-based FET device is influenced by the increased number of carriers in the channel. In other words, the *V*_g,min_ will be shifted leftwards and the extent of the shift increases with the increasing thickness of the membrane from 0.01 nM to 10 μM. In order to verify the proposed model, the effect of membrane thickness will be assumed and *G*_LP_ is modified as a function of electric charge (*Q*_LP)_ and membrane thickness as follows:

(7) $$G_{\mathrm{LP}}=G_{\mathrm{Neutral}}+\left(\alpha Q_{\mathrm{LP}}+\beta L_{\mathrm{LP}}\right)$$

where (*β*) and *L*_LP_ are the thickness parameter and thickness of the adsorbed lipid bilayer, respectively. In the non-saturation region, the GFET conductance model is involved as a result of gate electrical energy and the perfect conductance-voltage related to the graphene channel of the GFET device, which leads to the modified conductance as:

(8) $$\begin{array}{l} G_{\mathrm{LP}}=\left(\frac{3q^{2}{\left(3\pi a^{3}t^{3}k_{\mathrm{BT}}\right)}^{\frac{1}{2}}}{\mathrm{hl}}\left[{ℑ}_{\frac{-1}{2}}\left(\eta \right)+{ℑ}_{\frac{-1}{2}}\left(-\eta \right)\right]\right)\\ \;+\left(\alpha Q_{\mathrm{LP}}+\beta L_{\mathrm{LP}}\right) \end{array}$$

**Figure 8:**
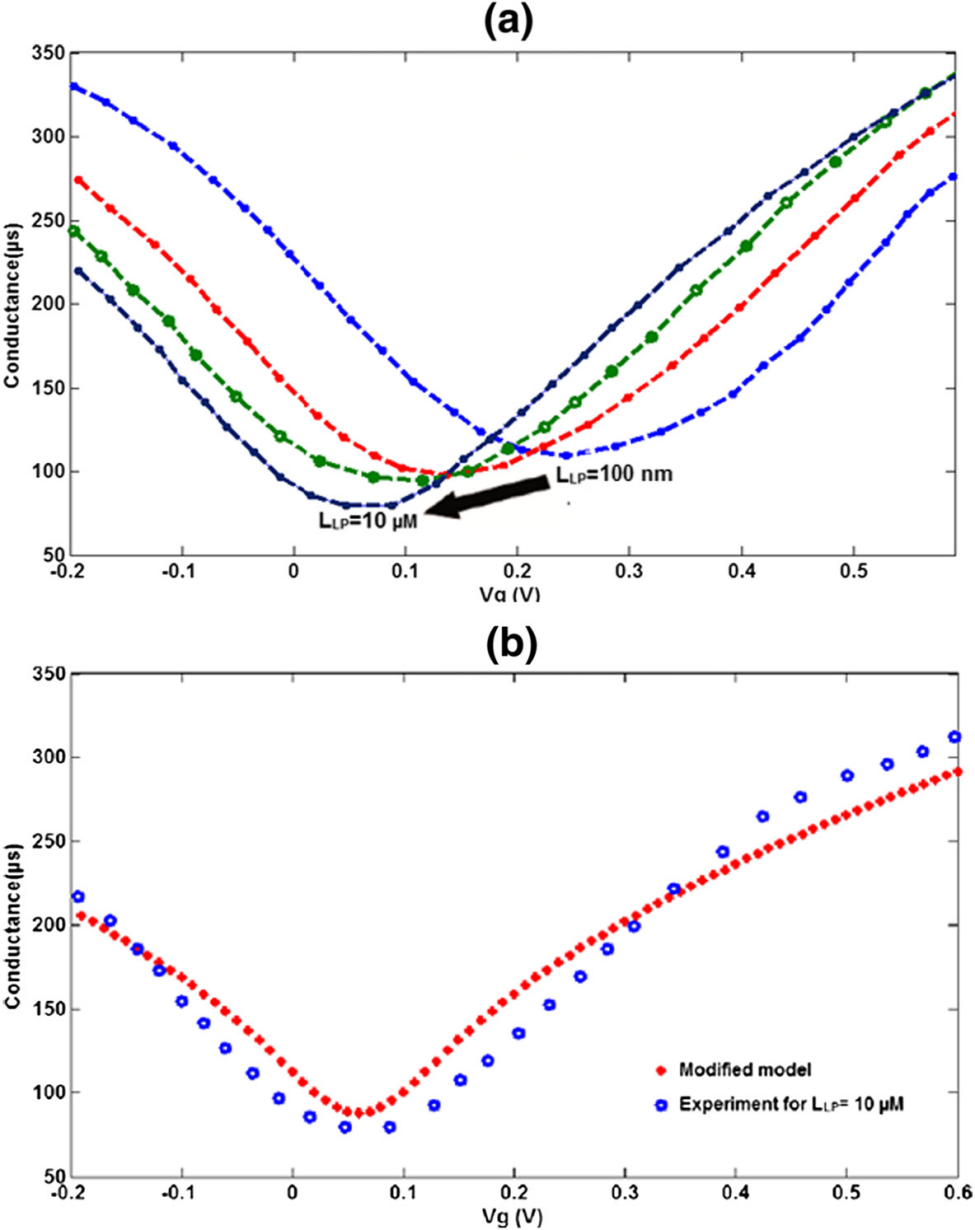
**Extracted experimental data for membrane thickness effect and *G*-*V***_**g **_**characteristic of proposed conductance model. (a)** Extracted experimental data for membrane thickness effect of biomimetic membrane-coated graphene biosensor. **(b)***G*-*V*_g_ characteristic of proposed conductance model with experimental data [10] for 10-μM membrane thickness.

In Figure 8b, all the theoretical *G*_LP_-*V*_g_ characteristics of graphene-based GFET with *L*_LP_ = 10 μM are plotted. Comparing Figures 8a and b, it can be seen that the biomimetic membrane-coated graphene biosensor model according to the suggested parameters (*α* and *β*) indicates the same trends as those reported by [10]. In both the experimental and theoretical data, there is a clear shift in *V*_g,min_ with increasing membrane thickness. Comparison of the experimental data depicted with the theoretical data in Figure 8 shows that a 10 μM membrane thickness caused a 10-meV shift in *V*_g,min_.

In the suggested model, differently charged lipid bilayers and membrane thicknesses are demonstrated in the form of *G*_LP_ and *L*_LP_ parameters, respectively, in agreement with the reported data which is shown in Table 1. The *V*_g,min_ did not shift further at greater membrane thicknesses due to the saturation current density of the injected carrier concentration by the charged lipid bilayer.

**Table 1:** **Different *Q***_**LP **_**and *L***_**LP **_**values with *V***_**g,min **_**changes**

	**V**_**g,min **_**(V)**
Q_LP_	
Neutral	0.11
Negatively	0.29
Positively	-1.1
L_LP_	
10 nm	0.24
0.1 μm	0.135
1 μm	0.09
10 μm	0.045

According to the saturation region of the presented conductance model and given that g_m,min_ belongs to the graphene-based biosensor, the control parameter with respect to the iteration method is suggested as:

(9) $$\beta =l_{1}e^{-l_{2}L_{\mathrm{LP}}}$$

where *l*_1_ = 0.4157 and *l*_2_ = -0.543. In addition, *α* for the neutrally, negatively, and positively charged membrane is assumed to be 0, 1, and -1, respectively. Consequently, the justified model for the interaction of charged impurity and the consequence of charged lipid membranes in a biomimetic membrane-coated graphene biosensor is proposed as

(10) $$\begin{array}{l} G_{\mathrm{LP}}=\left(\alpha Q_{\mathrm{LP}}+L_{\mathrm{LP}}l_{1}e^{-l_{2}L_{\mathrm{LP}}}\right)\\ \;+\left(\frac{3q^{2}{\left(3\pi a^{3}t^{3}k_{\mathrm{BT}}\right)}^{\frac{1}{2}}}{\mathrm{hl}}\left[{ℑ}_{\frac{-1}{2}}\left(\eta \right)+{ℑ}_{\frac{-1}{2}}\left(-\eta \right)\right]\right) \end{array}$$

The proposed model, coupled with the experimental data, is shown in this work to confirm that the conductivity of the graphene-based biosensor is changed by the electric charge and membrane thickness of the lipid bilayer. In a nutshell, electrolyte-gated graphene field-effect transistor structure was used after chemical vapor deposition (CVD) as the electrical transduction stage because of its high electrical conductivity, optical transparency, and large area, given the likelihood of manufacturing a dual-mode optical and electrical detection system for detecting the changes of membrane properties. Based on what has been discussed, one could firmly claim that, in response to changes of the charged lipid membranes and charges of biomimetic membranes of different thicknesses, a significant shift in *V*_g,min_ of the ambipolar FET occurs due to the electronic devices on both the n-doping and p-doping materials.

## Conclusion

The emerging potential of nanostructured graphene-based biosensors in the highly sensitive and effective detection of single-base polymorphism or mutation, which is thought to be the key to diagnosis of genetic diseases and the realization of personalized medicine, has been demonstrated. In a lipid bilayer-based biosensor, the graphene carrier concentration as a function of the lipid bilayer can be modeled. In this research, the total conductance of graphene as a function of the electric charge (*Q*_LP_) and thickness of the adsorbed lipid bilayer (*L*_LP_) is presented. A dramatic decrease in the minimum conductance related to the gate voltage (*V*_g,min_) by both changing the electrical charge from negative to positive and decreasing the lipid thickness has been reported. In the presented model, the *V*_g, min_ variation based on the adopted experimental data as an electrical detection platform is considered and the sensor control parameters are defined. The presented model confirms the reported experimental data and in addition facilitates the employment of alpha and beta as biosensor control parameters to predict the behavior of graphene in graphene-based biosensors.

## Competing interests

The authors declare that they have no competing interests.

## Authors’ contributions

MJK wrote the manuscript and contributed to the analytical modelling of the presented FET via MATLAB software. Dr. FKCh and Dr. MTA revised the manuscript and coordinated between all the contributors. HKFA, MR, and AH organized the final version of the manuscript. All authors read and approved the final manuscript.
